# Lactine as an Article of Nutrition

**Published:** 1853-08

**Authors:** 


From the Transactions of College of Physicians.

*Lactine as an Article of Nutrition.*

Dr. Ruschenberger stated that he had recently made trial of lactine, or sugar of milk, in a case of extreme irritability of the stomach. occuring after a profuse loss of blood from menorrhagia. The stomach would not retain any even of the mildest forms of nutriment adapted to the case. Having seen a suggestion in a western journal for the employment of lactine in place of cod-liver oil, and from its being tasteless, believing that it might possibly be tolerated by the stomach of his patient, Dr. R. directed her to take it, first, in small masses, to be gradually chewed and swallowed, and subsequently in larger quantities. It was without difficulty retained, and the patient under its continued use improved greatly—gaining strength, and the stomach resuming its functions.

The suggestion in a western journal, referred to in the above article, was found, doubtless, in the report published in this journal of a discussion in the Cook Co. Med. Society, upon a valuable paper upon this subject by Dr. J. H. Bird. (EDS.)

